# Thermal Stability and Flame Retardancy of Rigid Polyurethane Foam Composites Filled with Phase-Change Microcapsule

**DOI:** 10.3390/ma17040888

**Published:** 2024-02-15

**Authors:** Qing Cao, Qinggui Cao, Lujie Zhou, Kai Yu

**Affiliations:** 1 College of Safety and Environmental Engineering, Shandong University of Science and Technology, Qingdao 266590, China; caoqing526@163.com (Q.C.); skd995954@sdust.edu.cn (L.Z.); 2 Department of Chemical Engineering and Safety, Shandong University of Aeronautics, Binzhou 256600, China

**Keywords:** phase-change microcapsule, RPUF, chitosan, flame retardant

## Abstract

The flammability of rigid polyurethane foam (RPUF) limits its application. A new type of chitosan phase-change microcapsule (CS/PCM) was successfully prepared by the condensation method with chitosan and gum acacia as the wall material and paraffin as the core material. CS/PCM was introduced into RPUF composite material as filler to improve the thermal and flame-retardant properties of polyurethane. The morphology, structure, thermal properties and flame retardancy of the materials were characterized by scanning electron microscopy (SEM), X-ray diffractometer (XRD), thermogravimetric (TG) analysis, differential scanning calorimetry (DSC) and cone calorimetry. It is found that when the CS/PCM content is 30 wt%, the latent heat of phase transition of RPUF-30 is 12.308 J/g, the limiting oxygen index (LOI) is 26.1% and the fire risk is reduced. The flame-retardant mechanism shows that the barrier effect provided by chitosan plays an important role in effectively blocking the transfer of heat and combustible gas, and improving the flame-retardant property of the composite. This paper provides a new idea for the application of CS/PCM in RPUF.

## 1. Introduction

Polyurethane (PU) materials have become the most complex and expansive category in the polymer world due to their wide range of kinds and features. A key position in the worldwide rubber and plastic industries is held by the PU sector. Rigid polyurethane foam (RPUF) is one of these branches and has many excellent properties [1,2]. However, RPUF is a flammable material, and toxic gases will be emitted from the combustion chamber, accompanied by strong olfactory stimulation. At the same time, heat will be released during the synthesis reaction of RPUF, which will cause heat accumulation when used in large quantities, which is easy to cause fire and poses a great threat to people’s lives and safety [3,4]. RPUF is an indispensable material in people’s lives and production, and its application fields are very broad. It has important applications in automotive, mining, machinery, waterproof materials, energy-saving, light industry and aerospace [5,6,7]. Therefore, improving the flame retardancy of RPUF and reducing its reaction temperature are the problems that need to be solved in the application of RPUF.

With the rise of phase-change materials, more and more materials are combined with phase-change materials and have certain functions in phase-change energy storage [8]. Due to the effect of phase-change material energy storage, the phase-change material is applied to RPUF to absorb the heat of the RPUF reaction, which is the reaction of polyether polyols with isocyanates when polyurethanes are formed, and reduce the reaction temperature of the composite. However, the phase-change enthalpy of solid–solid phase=change materials is low, and solid–liquid phase-change materials and solid–gas phase-change materials are easy to leak when the phase change occurs during the phase-change process, which leads to the inability of polyurethane to directly combine with phase-change materials. Therefore, the leakage problem can be solved by making the phase-change material into a phase-change microcapsule (PCM), and then the PCM is added to RPUF to improve the flame retardancy of RPUF and reduce the reaction temperature of RPUF.

For the study of phase-change microcapsule rigid polyurethane foam (PCM/RPUF) composite materials, scholars have conducted the following research. Borreguero, Serrano et al. [9,10] studied the effects of the addition of PCM on the latent heat, structure, mechanical properties and thermal conductivity of polyurethane foam. Wang Xuechen et al. [11] prepared a phase-change capsule of melamine resin/n-octadecane and added it to polyurethane soft foam to analyze the heat treatment conditions of the PCM and its effects on the structure, compression properties and component viscosity of polyurethane foam. You et al. [12,13] prepared microcapsules containing melamine and applied them to the thermal insulation flexible foam of PU foam. The heat storage capacity and thermal regulation capacity of foam increased with the increase in microplate content. Niu et al. [14] used in situ polymerization to insert 4,4′-methylene diphenyl diisocyanate (MDI)-grafted carbon nanotubes (M-CNT) into phase-change material microcapsules (MPCM) (M-C-MPCM), and the enthalpy of M-C-MPCM was 90.28 J/g. Cone calorimeter analysis of RPUF containing M-C-MPCM shows that MDI and carbon nanotubes can work together to inhibit heat and smoke during combustion.

The research of scholars mainly focuses on the structure, mechanical properties and heat storage properties of microcapsules/polyurethane foam, but there are few studies on the flame-retardant properties of polyurethane foam after adding PCM. Therefore, a new type of PCM with certain flame-retardant properties was prepared in this paper, and the effect of PCM on the properties of RPUF was studied by adding CS/PCM to RPUF.

Paraffin wax (PW) is an important phase-change material that melts and solidifies at almost constant temperatures. PW has attracted wide interest in thermal management due to its low subcooling temperature, low cost and wide range of phase-transition temperatures [15]. Obviously, PW can be used to improve the thermal management performance of RPUF, but the direct introduction of PW will not only reduce the durability but also lead to PW leakage, which will adversely affect the flame retardance of the composite.

Chitosan (CS) is a natural polysaccharide polymer that is easily available and cheap. It has the characteristics of being non-toxic and harmless, relatively green environmental protection, easy biodegradation, no pollution to the environment, chemical stability, environmental friendliness and so on. Its molecular framework is rich in carbon and contains a certain number of hydroxyl and amino groups of side chain groups. In the process of thermal decomposition, carbonization in the polymer can hinder the combustion process, and at the same time, non-toxic and non-corrosive non-flammable gases such as CO_2_, NH_3_ and N_2_ will be released, thus playing a flame-retardant role, which is an ideal capsule wall material [16,17].

In this paper, CS/PCM was prepared by using CS and gum acacia as the wall material, and using paraffin as the core material by the condensation method. The effects of CS/PCM on the structure, phase transformation, thermal stability, and flame retardancy of RPUF were systematically studied by introducing CS/PCM into RPUF, and the flame retardancy mechanism was discussed. This paper provides a simple, low-cost and efficient method that provides a feasible solution for the thermal management application of CS/PCM in RPUF, provides a new idea for the functionalization of RPUF composite materials and promotes its application in construction, mining and other fields.

## 2. Materials and Methods

### 2.1. Materials

CS and glacial acetic acid, both analytically pure, are provided by Sinopharm Group Chemical Reagent Co., Ltd. (Shanghai, China). Glutaraldehyde and petroleum ether, both analytically pure, are supplied by McLean Technologies Ltd. Gum Arabic, chemically pure, is provided by Yusuo Chemical Technology Co., Ltd. (Qingdao, China).

Polyether polyol R4110 is supplied by Good Chem Ltd. (Hongkong, China) Catalyst A-33, dichlorofluoroethane, silicone oil, crosslinking agent DMP-30 and polyisocyanate are all provided by Shanghai Baiang Chemical Technology Co., Ltd. (Shanghai, China) The above reagents are industrial-grade.

### 2.2. Preparation of Composite Materials

#### 2.2.1. Preparation of Phase-Change Capsules

**Figure 1:**
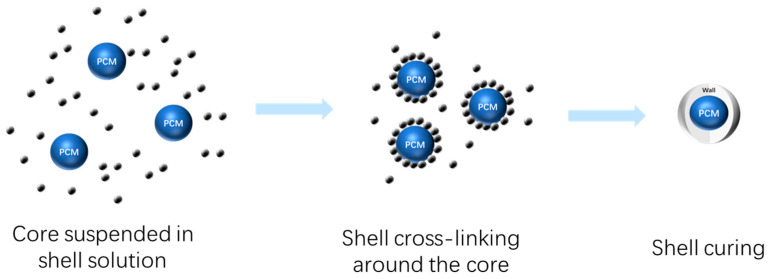
The schematic preparation procedure of CS/PCM.

The core material was PW, and the wall material was gum Arabic and chitosan. The mass fraction of gum Arabic in distilled water was 4%, and the mass fraction of chitosan in distilled water was 2%. Amounts of 4 g of paraffin wax and 4 g of gum Arabic were placed in a 250 mL three-mouth flask, melted at 65 °C and stirred at 600 rpm. Then, chitosan solution and 10% glacial acetic acid solution were added to the three-mouth flask. The pH of the solution was adjusted to 4.5, and after the condensation reaction for 20 min, the temperature was lowered to 15 °C in the ice water bath. An amount of 0.5 mL of glutaraldehyde was added to the solution and cross-linked for curing for 30 min. The temperature was raised to 40 °C, and the reaction was continued for 60 min. After filtration, the petroleum was washed with ether. After drying at 40 °C for 12 h, CS/PCM was obtained. The preparation process of CS/PCM is shown in Figure 1.

#### 2.2.2. Preparation of Composite Materials

Using polyether polyol R4110 and polyisocyanate PM200 as the base material, chitosan phase-change microcapsule rigid polyurethane foam (CS/PCM/RPUF) composites were prepared by adding CS/PCM, catalyst, foaming agent, foam stabilizer and crosslinking agent. Polyether polyol, catalyst A-33, crosslinking agent DMP-30, blowing agent, CS/PCM and surfactant were added to the paper cup. Stir well with an electric mixer on low speed at room temperature. Isocyanate was added and mixed well with an electric mixer. It was observed when bubbles were emitted and the bubble body was white; stirring was immediately stopped and then left for the mixture to foam and expand. The addition amount of CS/PCM was 5 wt%, 10 wt%, 15 wt%, 20 wt%, 25 wt% and 30 wt% of the mass of polyether polyol, respectively. The composites after adding CS/PCM are denoted as RPUF-5, RPUF-10, RPUF-15, RPUF-20, RPUF-25 and RPUF-30.

### 2.3. Characterization

The latent heat and temperature of phase change were measured by a differential scanning calorimeter (TA, 250, New Castle, DE, USA). Under N_2_ protection, the temperature ranged from 20 °C to 150 °C, and the heating rate was 10 °C/min. Fourier transform infrared spectroscopy (Thermo Nicolet380, Markham, ON, Canada) was used to measure the functional groups of samples in the range of 4000–400 cm^−1^.The samples were characterized by a RIGAKU smartlab (Tokyo, Japan) X-ray diffractometer (XRD) with a Cu target, an operating voltage of 40 kV, a current of 40 mA, a scanning range of 5–55°, a step angle of 0.08° and a sampling time of 0.5 s. The sample residue was characterized by a Thermo Scientific DXR Raman spectrum analyzer, and the scanning wave number range was 500–1200 cm^−1^. Scanning electron microscopy (SEM) (JSM-6510LV, JEOL Ltd., Tokyo, Japan) was used to test the surface morphology of samples. Thermogravimetric analysis (STA 449 F3, NETZSCH, Wunsiedel, Germnay) is used to test the thermal stability of samples. Test conditions: in the N_2_ atmosphere, temperatures rise from 30 °C to 700 °C at a rate of 10 °C/min. The thermal behavior of foam during combustion was measured by a cone calorimeter (Motis CCT). The SH5706A (Sunho, Guangzhou, China) oxygen index meter is used to find the limiting oxygen index (LOI) of a sample with dimensions 120 mm × 13 mm × 13 mm. CONE test procedures are carried out in accordance with ISO 5660 [18]. The test conditions were heat flux of 35 kW/m^2^, horizontal position of the sample and sample size of 100 mm × 100 mm × 25 mm. The thermal conductivity was tested by a PBD-12-4P (JiuTian Heat Insuaion Material Co., Ltd., Hejian, China) flat plate thermal conductivity tester with sample size 150 mm × 150 mm × 10 mm.

## 3. Results and Discussion

### 3.1. Surface Topography Analysis

**Figure 2:**
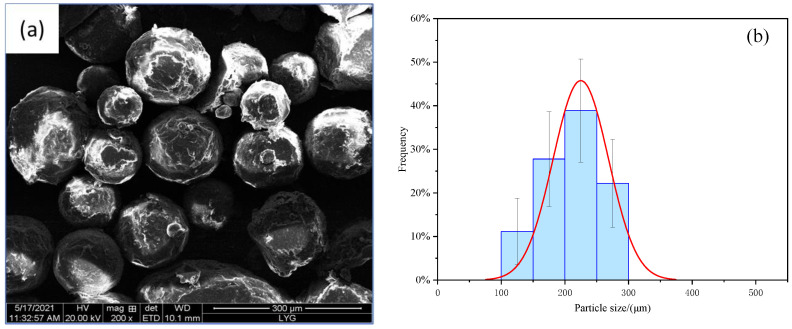
(**a**) SEM of CS/PCM and (**b**) Particle Size of CS/PCM.

**Figure 3:**
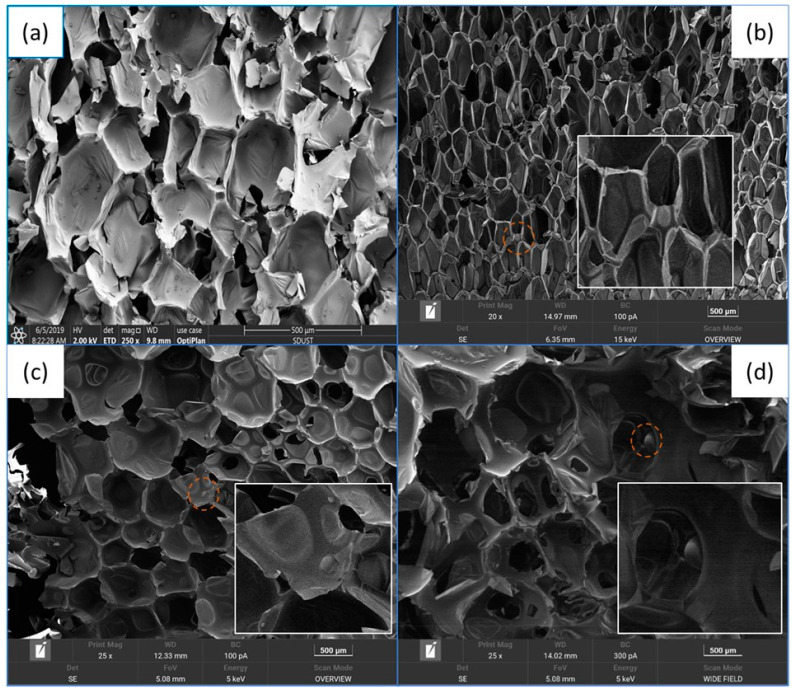
SEM of (**a**) RPUF, (**b**) RPUF−10, (**c**) RPUF−20 and (**d**) RPUF−30. The red circles are the positions of the PCM corresponding to the original image and the enlarged image.

**Figure 4:**
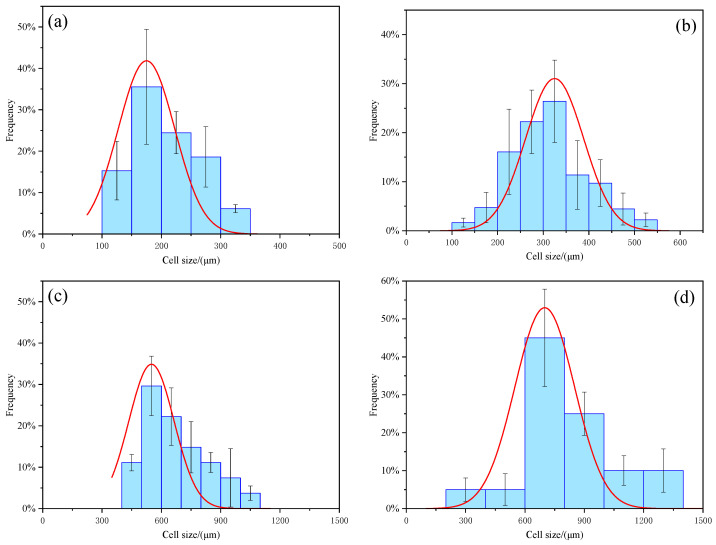
Cell size distribution of (**a**) RPUF, (**b**) RPUF−10, (**c**) RPUF−20 and (**d**) RPUF−30.

The SEM of CS/PCM and RPUF after adding CS/PCM is shown in Figure 2a and Figure 3. The particle size distribution of CS/PCM and the cell size distribution of RPUF after adding CS/PCM are shown in Figure 2b and Figure 4.

As can be observed from the figure, the particle size of CS/PCM ranges from 100 μm to 300 μm, following a normal distribution. RPUF composite with CS/PCM showed the state of bubble holes, and the distribution of bubble holes was arranged. With the increase in CS/PCM addition, the cell size of RPUF increased gradually. When CS/PCM participated in the synthesis of RPUF composite material, it was coated by the material, and most of the capsules did not leak out, obviously. When the addition of CS/PCM was 30 wt%, the arrangement of the cell structure was disrupted, the cell wall became thin and there was obvious tearing at the boundary of the sample. This is because a large number of flame retardants that do not participate in the polyurethane foaming reaction affect the result of the foaming reaction, resulting in increased pore structure spacing.

### 3.2. Fourier-Transform Infrared (FT-IR) Spectra Analysis

**Figure 5:**
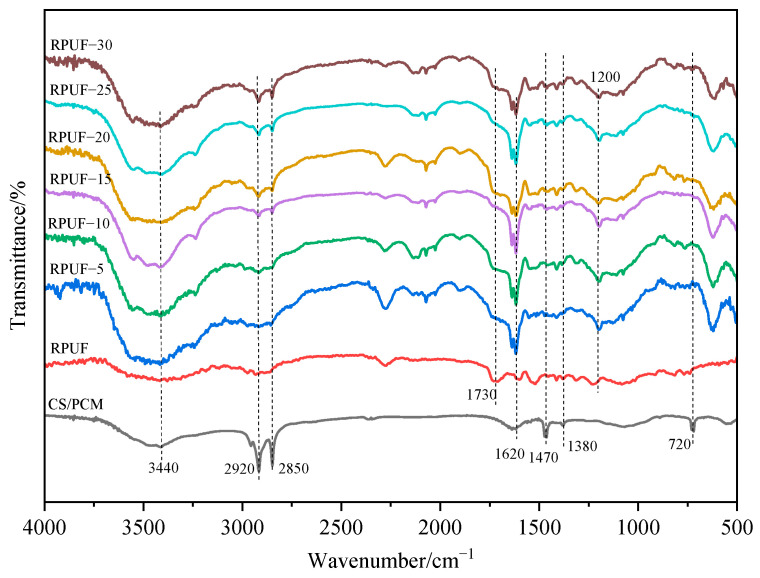
FT−IR of CS/PCM and RPUF.

FT-IR images of CS/PCM and RPUF with different contents of CS/PCM are shown in Figure 5. It can be seen from the figure that the absorption peak of CS/PCM at 720 cm^−1^ is the methylene in-plane oscillating vibration absorption peak. The absorption peaks at 1380 cm^−1^ and 1470 cm^−1^ belong to the bending vibration absorption peaks of methyl CH_3_ and methylene -CH_2_ [19,20]. The absorption peaks of RPUF near 1200 cm^−1^ and 1730 cm^−1^ are the stretching vibration peaks of the C-O and C=O bonds of carbamate in RPUF, respectively [21,22]. The absorption peak strength of the C=N stretching vibration increases near 1620 cm^−1^. The absorption peaks at 2850 cm^−1^ and 2920 cm^−1^ are the stretching vibration absorption peaks of methyl −CH_3_ and methylene −CH_2_, respectively [23,24]. A large and wide peak appears near 3440 cm^−1^, which is caused by the bonding of N−H and −OH bands through hydrogen bonding [25]. RPUF with CS/PCM has characteristic peaks of CS/PCM and pure RPUF. The intensity of each characteristic peak increased with the addition of the CS/PCM. These peaks indicate that the addition of CS/PCM to RPUF does not change the main structure of the RPUF.

### 3.3. XRD Analysis

**Figure 6:**
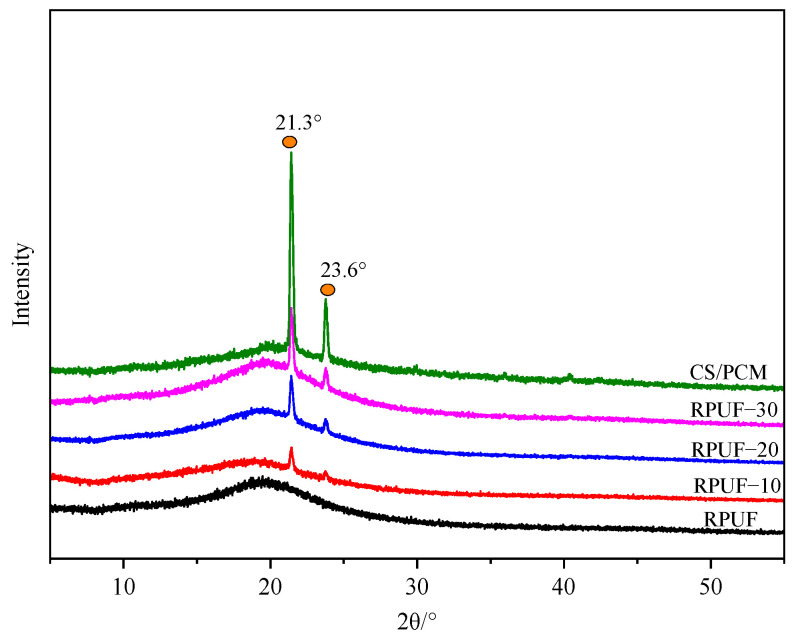
XRD of CS/PCM and RPUF.

The structure of CS/PCM and RPUF with different CS/PCM contents was analyzed using an X-ray diffractometer, as shown in Figure 6. CS/PCM has two sharp diffraction peaks at 2θ = 21.3° and 2θ = 23.6°, corresponding to the (110) and (200) crystal planes, respectively [15]. This is because CS/PCM contains PW, which has a crystal structure. In the RPUF spectrum, there is only a wide dispersion peak near 2θ = 20°, indicating that RPUF has an amorphous structure. The RPUF spectra with CS/PCM added all have the diffraction peak of CS/PCM and the dispersion peak of RPUF. However, the diffraction peak is not as strong as the CS/PCM peak, which means that the composite material only has CS/PCM and no other substances are created.

### 3.4. Thermogravimetric (TG) Analysis

**Figure 7:**
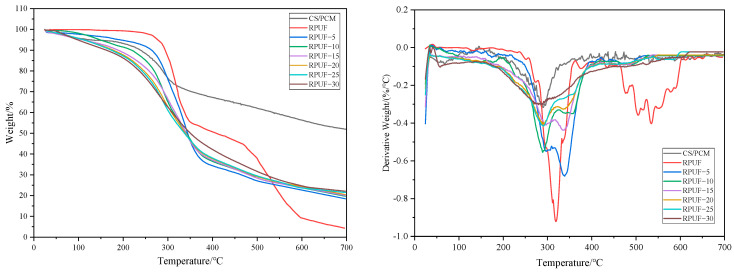
TG and DTG of CS/PCM and RPUF.

The TG and differential thermal gravity (DTG) tests of CS/PCM and RPUF with various amounts of CS/PCM are shown in Figure 7. In an N_2_ atmosphere, the CS/PCM has an obvious rapid pyrolysis stage. The main reason for the weight loss near 100 °C is the evaporation of water on the surface of the capsule. Rapid thermogravimetry of CS/PCM works best at temperatures between 200 °C and 350 °C. This is because paraffin wax breaks down at these temperatures, and gum Arabic and chitosan also break down.

The weight loss process of RPUF composites with different contents of CS and PCM can be roughly divided into three stages. The first stage is mainly the weight loss of a part of the phase-change capsule core material in the composite material. The second stage is the degradation of the remaining core material and the hard chain segment of RPUF. The slope of the TG curve changes obviously, and the weight loss phenomenon intensifies. The third stage is mainly the thermal degradation of the wall material of PCM and the soft chain segment of RPUF [26,27].

The starting thermogravimetric temperature of RPUF composites with different amounts of CS/PCM is around 50 °C in the first stage. The weight loss curve changes slowly, and as more CS/PCM is added, the weight loss rate slowly increases. In the second stage, with the addition of CS/PCM, the initial temperature decreases continuously. The starting temperature of the RPUF with 5 wt% CS/PCM was 258 °C, and the ending temperature was 369 °C. The starting temperature of RPUF with 30 wt% CS/PCM is 203 °C, and the ending temperature is 348 °C. At this stage, the weight loss rate is fast, and the core material of the phase-change capsule is basically pyrolytic in the first half stage. The weight loss rate of the third stage is basically the same, which is mainly the thermal degradation of the remaining part of the phase-change capsule wall material and the soft chain segment in RPUF.

### 3.5. DSC Analysis

**Figure 8:**
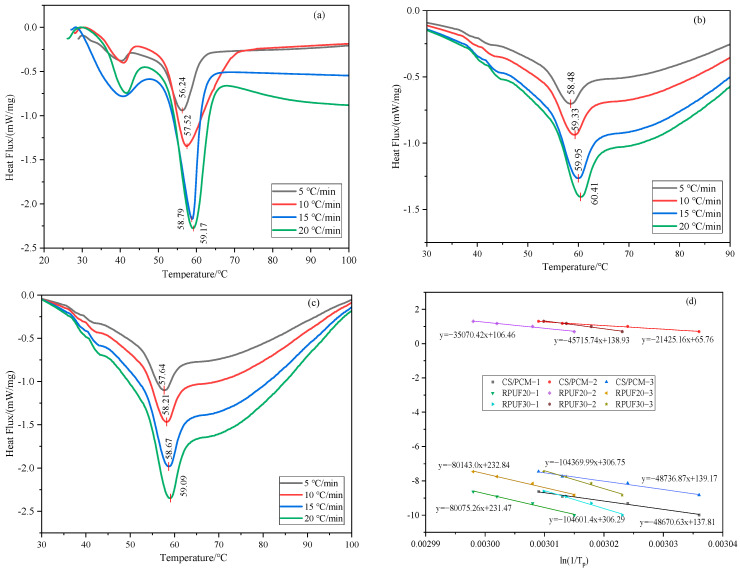
DSC and activation energy fitting diagrams of CS/PCM and RPUF at different heating rates. (**a**) DSC of CS/PCM; (**b**) DSC of RPUF −20; (**c**) DSC of RPUF−30; (**d**) activation energy fitting diagram (1 for Kissinger method, ordinate ln(β/T_p_^2^); 2 for Ozawa’s method, ordinate ln(β); 3 for Starink method, ordinate ln(β/T_p_^1.8^)).

DSC analysis was performed on CS/PCM, RPUF-20 and RPUF-30, respectively, at four different heating rates of 5 °C/min, 10 °C/min, 15 °C/min and 20 °C/min, as shown in Figure 8a–c.

The DSC curve of pure RPUF does not have a melting peak or endothermic peak and does not have a latent heat of phase transition. The RPUF composite with CS/PCM has an obvious endothermic peak. The graph shows that as the amount of CS/PCM added rises, the heat absorption peak of RPUF gradually grows, as does the melting peak area. The starting temperature for the phase transition also rises, and so do the phase transition peak temperature and melting peak area. According to the DSC curve, the latent heat of the RPUF phase transition with the addition of 30 wt% CS/PCM is 12.308 J/g. The peak value of pyrolysis in pure RPUF is superimposed with the peak value of the phase transition capsule. After adding CS/PCM, the peak phase transition temperature of the composites increased. This is due to the fact that when the temperature rises, a part of the heat is used for the degradation of the material.

The Kissinger Formula (1), Ozawa Formula (2) and Starink Formula (3) were used to calculate the phase transition activation energy and the pre-exponential factor [28].

(1) $$\ln\left(\frac{\beta }{T_{p}^{2}}\right)=\ln\frac{AR}{E}-\frac{E}{RT_{p}}$$

(2) $$\mathrm{lg}\beta =\ln\left(\frac{AE}{Rg\left(a\right)}\right)-2.315-0.4567\frac{E}{RT}$$

(3) $$\mathrm{lg}\left(\frac{\beta }{T^{1.8}}\right)=Cs-1.0037\frac{E}{RT}$$

where *A* is the pre-exponential factor, min^−1^ or s^−1^; *E* is the activation energy, kJ/mol; *R* is the universal gas constant, with a value of 8.314 J/(mol∙K); *T* is the thermodynamic temperature, K; *β* is the temperature rise rate, K∙min^−1^; and *T_p_* is the peak phase transition temperature of the composite at the heating rate, K. According to the apparent activation energy (Ea) obtained by the Kissinger formula method, the activation energy values calculated by the three methods are shown in Table 1.

It can be seen from Table 1 that RPUF with CS/PCM added has greater activation energy than CS/PCM alone. The reason for this is that adding composite materials slows down the molecular thermal diffusion movement of CS/PCM, which makes the activation energy go up. With the increase in CS/PCM addition, the activation energy of RPUF increased gradually. This may be due to the fact that during the preparation of composite materials, a large amount of composite materials are attached to the surface of CS/PCM, which hinders the thermal movement of phase-change material molecules. At the same time, the activation energy required for phase change also increases with the increase in CS/PCM content, thus increasing the influence on the phase-change temperature and latent heat of composite materials.

Figure 8d shows that the activation energies of the different samples calculated by the three methods tend to fit together well. The fitting results are close to 0.99, which means that the test data are correct and reliable [28].

### 3.6. Thermal Conductivity Evaluation and Analysis

**Figure 9:**
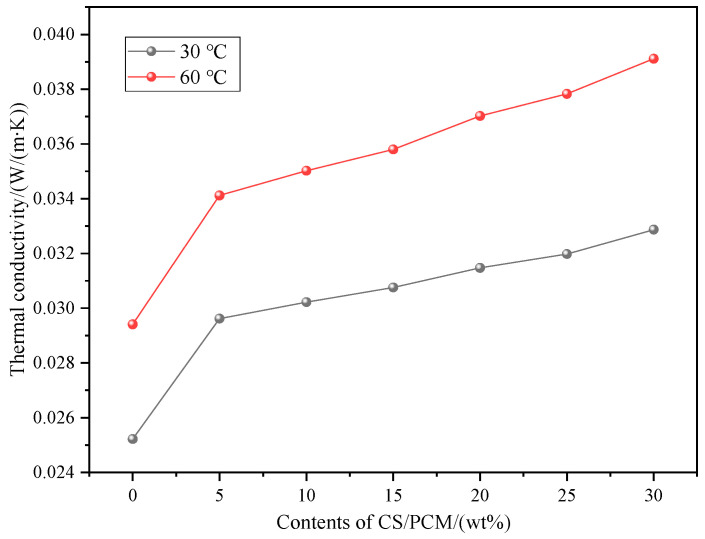
Thermal conductivity of RPUF with different contents of CS/PCM.

The thermal conductivity of RPUF with different contents of CS/PCM at 30 °C and 60 °C is shown in Figure 9. It can be seen from the figure that the thermal conductivity of RPUF composites measured at 30 °C and 60 °C increased with the addition of CS/PCM. When the content of CS/PCM is the same, the thermal conductivity of RPUF composites at 60 °C is greater than that at 30 °C. When 20 wt% CS/PCM was added at 60 °C, the thermal conductivity of RPUF composite was 0.03702 W/(m·K), which was 17.63% higher than that when 20 wt% CS/PCM was added at 30 °C, and 25.87% higher than that of pure RPUF. This is mainly because the addition of CS/PCM increases the foaming ratio of the composite material, fills the internal pores of the composite material and increases the thermal conductivity. With the increase of temperature, the thermal motion of the molecules inside the material increases, and the heat transfer increases, so the thermal conductivity of the composite material increases at 60 °C.

### 3.7. Flame-Retardant Performance

**Figure 10:**
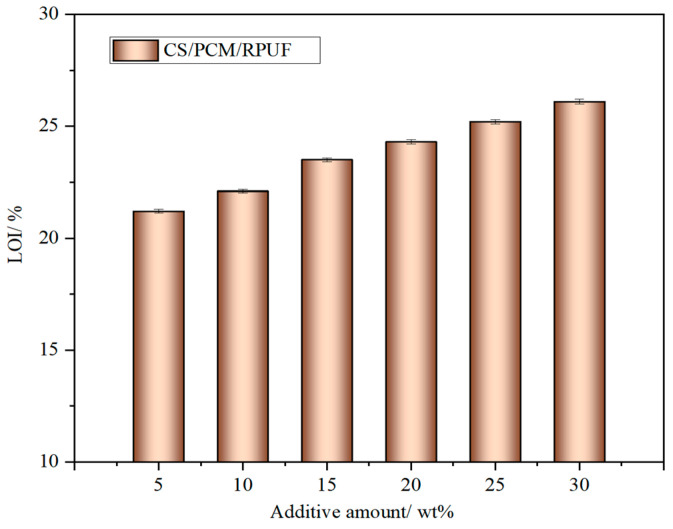
LOI of RPUF with different contents of CS/PCM.

In order to study the flame retardancy of the composites, an LOI test and a cone calorimetric test were carried out, respectively. LOI tests were conducted on RPUF composites with different contents of CS/PCM, and the results are shown in Figure 10. Pure RPUF is a Class B3 flammable material that burns readily at typical oxygen conditions and has a low LOI of only 19%. After adding CS/PCM, the LOI of RPUF composites is greater than 21%, and the LOI is improved, but the improvement value is relatively small. When the addition amount of CS/PCM is 30 wt%, the LOI of RPUF-30 is a maximum of 26.1%. RPUF-30 composite material is classified as a material of B2 grade.

**Figure 11:**
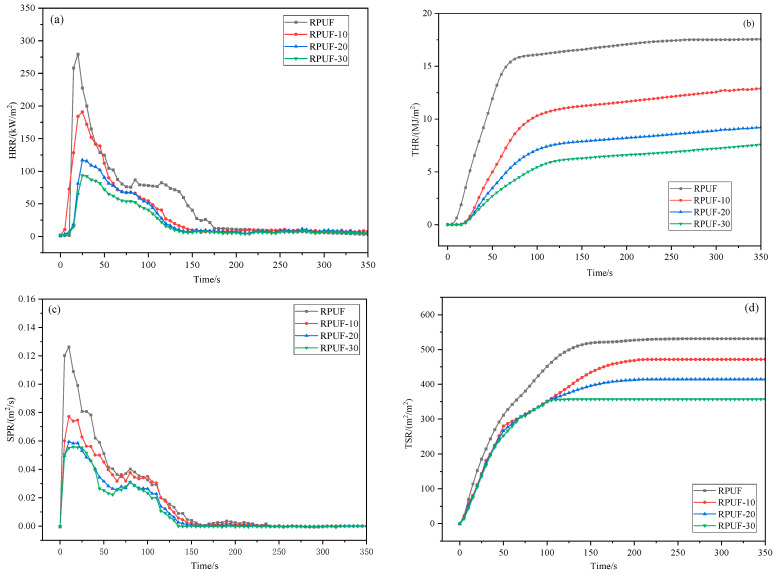
HRR, THR, MLR and SPR of RPUF with CS/PCM added. (**a**) HRR; (**b**) THR; (**c**) SPR; (**d**) TSR.

The change curves of heat release rate (HRR), total heat release (THR), smoke produce rate (SPR) and total smoke release (TSR) of RPUF with CS/PCM added are shown in Figure 11. The peak data of the cone calorimetry test are shown in Table 2.

After the addition of CS/PCM, the RPUF-30 took 25 s to peak heat release rate (PHRR) after combustion, which was reduced to 93.57 kW/m^2^ compared to the pure RPUF. The PHRR of RPUF-30 decreased by 66.5% compared with pure RPUF, and the peak smoke generation rate (PSPR) decreased by 55.8% compared with pure RPUF, and both PSPR and TSR gradually decreased. The RPUF with CS/PCM, the peak shape of the HRR curve, is no longer sharp, which is due to the addition of phase-change capsules, which absorb part of the released heat and slow down the release rate of heat. With the increasing amount of CS/PCM, the fire growth rate index (FIGRA = PHRR/tPHRR) of RPUF composites gradually decreased, and the fire safety improved. According to the study of Vahabi et al. [29], the flame retardant index (FRI) was calculated according to Formula (4). The FRI is less than 1, indicating that the flame-retardant performance of the material is poor. The FRI is between 1 and 10, and the material has good flame-retardant performance. Between 10 and 100, the material has excellent flame-retardant properties [30,31]. The FRI of RPUF-10, RPUF-20 and RPUF-30 is 2.0, 6.35 and 10.39, respectively, and the flame-retardant property of RPUF is significantly improved.

(4) $$\mathrm{FRI}=\frac{{\left[\mathrm{THR}\times \left(\frac{\mathrm{PHRR}}{\mathrm{TTI}}\right)\right]}_{\mathrm{RPUF}}}{{\left[\mathrm{THR}\times \left(\frac{\mathrm{PHRR}}{\mathrm{TTI}}\right)\right]}_{\mathrm{RPUF}/\mathrm{Flame}\;\mathrm{retardant}}}$$

### 3.8. Flame-Retardant Mechanism Analysis

**Figure 12:**
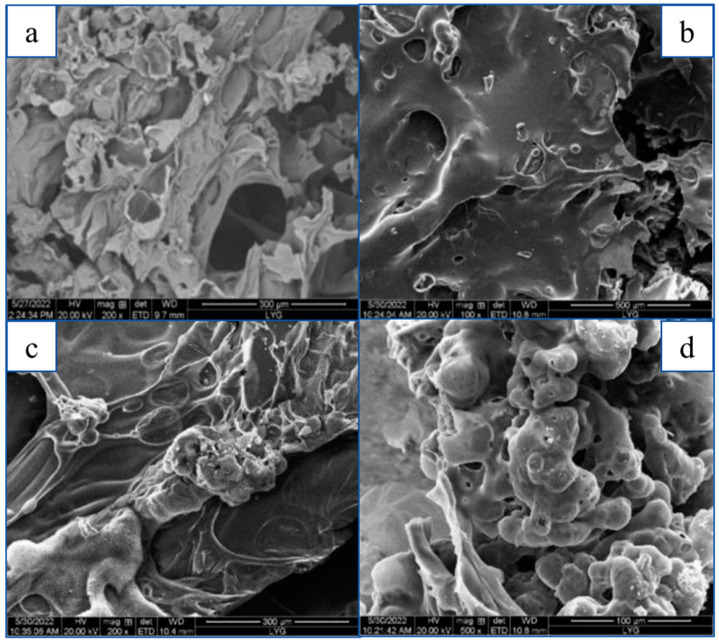
SEM of the burned RPUF residue supplemented with different contents of CS/PCM. (**a**) CS/PCM; (**b**) RPUF-10; (**c**) RPUF-20; (**d**) RPUF-30.

The SEM diagram of the residue after combustion of RPUF composites with different contents of CS/PCM added is shown in Figure 12. The residue after combustion was material from a cone calorimeter.

As can be seen from the figure, the surface of RPUF without CS/PCM is uneven, with cracks and gaps, and flame and heat transfer through the cracks, resulting in reduced fire resistance. The RPUF after the addition of CS/PCM was aggregated after combustion and decomposition, and the agglomeration phenomenon was obvious with the increase in the addition of CS/PCM, which was caused by the inclusion of gum Arabic in the wall material of the phase-change capsule. When the content of CS/PCM is low, the surface of the composite is rough after combustion, and there are cracks and cracks.

**Figure 13:**
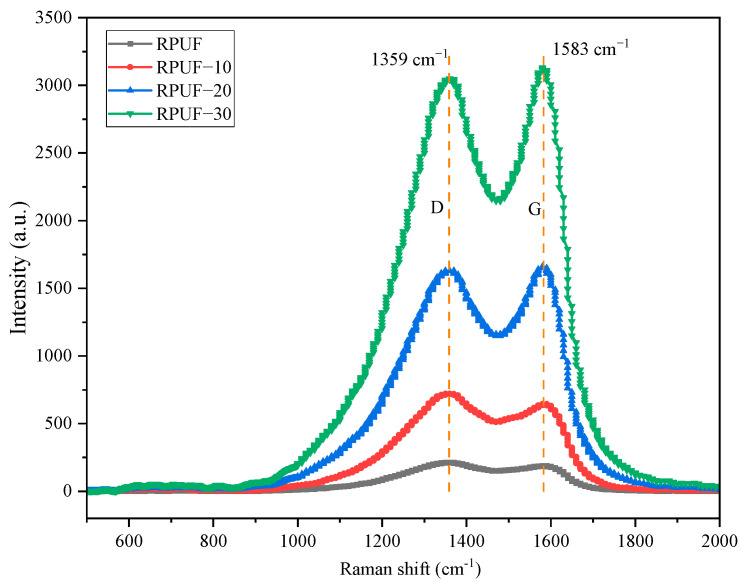
Raman spectra of the carbon residues after combustion test.

To learn more about how RPUF blocks flames after adding CS/PCM, Raman spectral analysis was performed on the residue after combustion of RPUF composites with different contents of CS/PCM added, as seen in Figure 13. The residue after combustion was material from a cone calorimeter, too. Ordered graphitized carbon (D-peak) and disordered carbon (G-peak) were detected near 1359 cm^−1^ and 1583 cm^−1^, respectively. ID/IG was calculated to evaluate the degree of graphitization of residual carbon after combustion. The lower the ID/IG value, the higher the degree of graphitization and the better the thermal stability [32]. As you can see from Figure 11, the ID/IG value of a pure RPUF is 1.13. The ID/IG value of the RPUF with 20 wt% CS/PCM added is 0.98. With the increase in CS/PCM addition, the D-peak and G-peak values of RPUF gradually increased, and the ID/IG values of RPUF gradually decreased. The findings show that adding CS/PCM can help make graphitized carbon, raise the level of graphitization, make the carbon residue more thermally stable, and lower the substrate mass loss and heat release.

**Figure 14:**
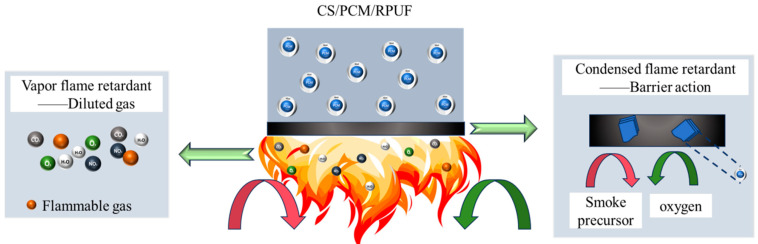
Flame-retardant mechanism of CS/PCM/RPUF.

The flame retardancy of CS/PCM/RPUF composites is mainly affected by CS/PCM. On the one hand, CS/PCM contains flammable core wax. With the increase in CS/PCM content, the paraffin content increases, which will have a significant negative impact on the flame-retardant properties of the composite. On the other hand, CS/PCM has the shell structure of chitosan, which has a positive effect on improving the flame retardancy of the composite. Therefore, a reasonable flame-retardant mechanism for RPUF composites containing CS/PCM was proposed, as shown in Figure 14. From the perspective of the vapor flame-retardant mechanism, when the shell of CS/PCM is attacked by flames, a large number of non-flammable gases (water, carbon dioxide and nitrogen dioxide) will be released, which can dilute combustible gases and oxygen in the vapor phase, thus improving the flame-retardant performance of RPUF composites [33,34]. In terms of the condensed phase, chitosan can form a C-N bond, promote the formation of a protective carbon layer and reduce the generation and emission of smoke [35]. The transfer of heat, combustible gas and oxygen is effectively blocked, and the flame-retardant property of the composite is improved.

## 4. Conclusions

In this study, CS was used to make flame-resistant CS/PCM, which were then mixed with RPUF. This made it possible to make RPUF composites that could control heat and put out fires. The results showed that the introduction of CS/PCM did not change the main structure of polyurethane. The CS/PCM/RPUF composites added with CS/PCM showed the state of bubble holes, the distribution of bubble holes was arranged and the maximum decomposition rate decreased. When the CS/PCM content is 30 wt%, the latent heat of RPUF-30 is 12.308 J/g, the oxygen index is 26.1%, the PHRR of the RPUF-30 is reduced by 66.5% and the fire risk is reduced. The flame-retardant mechanism shows that the capsule shell can not only release a large amount of non-flammable gas and dilute flammable gas but also promote the formation of a protective carbon layer, effectively block the transfer of heat, smoke and combustible gas and improve the flame-retardant property of composite materials. This paper provides a feasible solution for the thermal management application of CS/PCM in RPUF, provides a new idea for the functionalization of RPUF composite materials and promotes its application in construction, transportation and other fields.

## Figures and Tables

**Table 1:** Dynamic parameters of phase transformations of different samples.

Samples	*E_a_*_1_	*E_a_*_2_	*E_a_*_3_	*E_a_*	*lgA*	*R* ^2^
CS/PCM	404.65	390.03	403.70	399.46	64.54	0.99
RPUF-20	665.74	638.44	663.85	656.01	105.43	0.99
RPUF-30	869.66	832.23	864.53	855.47	138.04	0.98

*E_a_* is the average value of the three activation energies in kJ/mol, and *E_a_*_1_, *E_a_*_2_ and *E_a_*_3_ are the activation energies obtained using the Kissinger, Ozawa and Starink formulas, respectively.

**Table 2:** Cone calorimeter data of RPUF with different contents of CS/PCM.

Samples	TTI /(s)	PHRR /(kW/m^2^)	tPHRR /(s)	THR /(kJ/m^2^)	PSPR /(m^2^/s)	FIGRA	FRI
RPUF	2	279.58	20	17.57	0.1263	13.98	/
RPUF-10	2	190.83	22	12.85	0.0771	8.67	2.00
RPUF-20	3	126.01	25	9.21	0.0584	5.04	6.35
RPUF-30	3	93.57	25	7.58	0.0558	3.74	10.39

In the table, TTI is Time to Ignition; tPHRR is time required to reach peak heat release rate.

## Data Availability

The data used to support the findings of this study are available upon request from the corresponding author.
